# Precursor Phenomena of Barium Titanate Single Crystals Grown Using a Solid-State Single Crystal Growth Method Studied with Inelastic Brillouin Light Scattering and Birefringence Measurements

**DOI:** 10.3390/molecules23123171

**Published:** 2018-12-01

**Authors:** Soo Han Oh, Jae-Hyeon Ko, Ho-Yong Lee, Iwona Lazar, Krystian Roleder

**Affiliations:** 1 Department of Physics, Nano Convergence Technology Center, Hallym University, Chuncheon, Gangwondo 24252, Korea; soohanoh@naver.com; 2 Department of Materials Science and Engineering, Sunmoon University, Asan Chungnam 31460, Korea; hlee@ceracomp.com; 3 Institute of Physics, University of Silesia, ul. 75 Pułku Piechoty 1, 41-500 Chorzów, Poland; iwona.lazar@us.edu.pl (I.L.); Krystian.Roleder@us.edu.pl (K.R.)

**Keywords:** barium titanate, BaTiO_3_, Brillouin spectroscopy, acoustic property, precursor phenomena

## Abstract

The nature of precursor phenomena in the paraelectric phase of ferroelectrics is one of the main questions to be resolved from a fundamental point of view. Barium titanate (BaTiO_3_) is one of the most representative perovskite-structured ferroelectrics intensively studied until now. The pretransitional behavior of BaTiO_3_ single crystal grown using a solid-state crystal growth (SSCG) method was investigated for the first time and compared to previous results. There is no melting process in the SSCG method, thus the crystal grown using a SSCG method have inherent higher levels of impurity and defect concentrations, which is a good candidate for investigating the effect of crystal quality on the precursor phenomena. The acoustic, dielectric, and piezoelectric properties, as well as birefringence, of the SSCG-grown BaTiO_3_ were examined over a wide temperature range. Especially, the acoustic phonon behavior was investigated in terms of Brillouin spectroscopy, which is a complementary technique to Raman spectroscopy. The obtained precursor anomalies of the SSCG-grown BaTiO_3_ in the cubic phase were similar to those of other single crystals, in particular, of high-quality single crystal grown by top-seeded solution growth method. These results clearly indicate that the observed precursor phenomena are common and intrinsic effect irrespective of the crystal quality.

## 1. Introduction

Ferroelectricity is defined in terms of the appearance of the spontaneous polarization under a certain thermodynamic condition, which can be switched by applying an external electric field. Ferroelectric materials have been adopted in a wide range of applications, such as capacitors, memories, and piezoelectric devices. Among various ferroelectrics, perovskite-based oxide materials have attracted much attention due to their superior ferroelectric and electromechanical properties, and thus high potential in their applications. They are also promising materials from the viewpoint of fundamental physics because of their rich structural and dynamical properties during the ferroelectric phase transition [1,2].

Barium titanate (BaTiO_3_) is one of the most well-known ferroelectrics, where the molecular unit BaTiO_3_ comprises the primitive unit cell in the paraelectric cubic phase. BaTiO_3_ exhibits successive phase transitions from cubic to tetragonal at *T_C_* ≈ 132 °C, tetragonal to orthorhombic, and orthorhombic to rhombohedral phase upon cooling [1]. There has been intense debate on the nature of the ferroelectric phase transition of BaTiO_3_, whether its mechanism is displacive or order–disorder type. It was originally thought to be a displacive ferroelectric where the lowest transverse optic (TO) mode plays a central role during the phase transition [3]. However, many experimental results on BaTiO_3_ were presented favoring the viewpoint of the order–disorder phase transition. Part of these reports include the observation of diffuse X-ray scattering [4], incomplete softening of the soft TO mode near *T_C_* [5], deviation of the refractive index from high-temperature linearity [6], and first-order Raman scattering in the cubic phase [7]. It is now widely accepted that both the order–disorder and the displacive components contribute to the ferroelectric phase transition of BaTiO_3_ [8,9].

Special attention has been paid to the pretransitional or precursor phenomena in the paraelectric cubic phase of BaTiO_3_. Diffuse X-ray scattering suggests off-centered motions of Ti atoms along the <111> direction [4,10]. Early neutron scattering experiment also suggested the existence of a critical polarization fluctuations in the cubic phase based on the observed quasi-elastic scattering [11]. Local rhombohedral distortions were revealed using X-ray absorption spectroscopic studies [12]. Burns and Dacol attributed the anomalous behavior of the refractive index to the existence of precursor local polarizations in the paraelectric phase [6]. Precursor polar clusters are randomly-oriented polar regions with local symmetry-breaking while the macroscopic cubic phase is preserved with inversion symmetry. The precursor polar clusters are expected to manifest relaxational motions at high temperatures, which were evidenced via hyper-Raman scattering [13], terahertz dielectric response [14], and central peaks observed by both Raman and Brillouin scattering [15,16,17]. Moreover, broken inversion symmetry in the precursor clusters could be investigated and confirmed by other various experimental techniques, such as piezoelectric constant [18], birefringence [19,20], photon correlation spectroscopy [21], Raman spectroscopy [7,22,23], second harmonic generation [24], speckle measurements using a pulsed X-ray technique [25], thermal expansion [26], and elastic properties [27,28]. Additional relaxation modes were observed in the THz range in the ferroelectric tetragonal phase [29,30].

The precursor dynamics of BaTiO_3_ shows similarity with relaxor ferroelectrics in which the appearance of polar nanoregions (PNRs) begins at the so-called Burns temperature (*T_B_*) [31]. The *T_B_* of BaTiO_3_ was found to be ≈280 °C as determined by acoustic emissions [32] or ≈313 °C via resonance ultrasonic spectroscopy (RUS) [28]. Aktas et al. suggested that a local piezoelectric effect persists up to ≈340 °C investigated in terms of the resonance piezoelectric spectroscopy [33]. The precursor polarization is considered to be one of the origins for the large flexoelectricity of BaTiO_3_ in the paraelectric phase [34,35].

In spite of these extensive studies, the origin of precursor polarizations in the paraelectric phase is still not clearly understood. The universal scenario for the polar clusters based on the coupling between short-range structural instabilities and long-wavelength polar soft modes was proposed as an intrinsic effect [36,37]. First-principle calculations suggested the formation of eight minima along the <111> directions in the local free energy, which persist up to (*T_C_* + 400) °C in the paraelectric phase [38]. Anharmonic potential for the Ti vibrations was also reported by other studies [25]. In addition to these theoretical and experimental works where the precursor phenomena are considered as an intrinsic effect, the possibility of an extrinsic effect on the precursor phenomena needs to be addressed because the effect of impurities on the phase transition [39] or defect contribution to the pretransitional anomaly has been other important issues. Dislocations, extended defects, or surfaces may be the favorable regions where polar clusters can be formed easily [40].

In this study, we focus on the precursor phenomena of BaTiO_3_ single crystals grown using a solid-state single crystal growth (SSCG) method [41,42] and compare the results with those of our previous results on BaTiO_3_ single crystals grown using a top-seeded solution growth (TSSG) method [17]. SSCG method is a unique growth technology where a melting process is not required during the crystal growing. It has originally been developed for low-cost mass production. Because of this advantage and fast growth process, it has demerits such as relatively high level of defects, impurities, and air pores. Since the two kinds of crystals grown by different techniques exhibit substantial difference in the defect level (see Section 4. Materials and Method), systematic comparison between their properties is a good opportunity for investigating the effect of defect/impurities on the pretransitional phenomena of BaTiO_3_.

In the present study, we focus on the acoustic and birefringence properties in order to investigate precursor phenomena of BaTiO_3_. The acoustic anomalies have been studied using several experimental techniques, such as the Brillouin light scattering [16,17,43,44] and ultrasonic techniques including RUS [28,45]. All these studies show that substantial softening of certain acoustic modes occurs in the paraelectric cubic phase. For example, the mode frequency of the longitudinal acoustic (LA) phonon showed a substantial decrease in the paraelectric phase upon cooling. The hypersonic damping shows a significant increase on approaching *T_C_* from the high-temperature side. These previous studies showed that acoustic investigation is a good experimental tool for studying the dynamics of precursor polar clusters. In order to investigate acoustic properties of SSCG-grown BaTiO_3_, we apply Brillouin spectroscopy, which is one of the inelastic light scattering techniques in the low-frequency range below 1 THz and is thus a complementary technique to Raman spectroscopy [46,47,48]. This technique is also complementary to other ultrasonic tools that are useful to study ferroelectric and piezoelectric properties of various perovskites [49]. In addition to the acoustic anomalies, birefringence is a direct method [50] for probing non-centrosymmetric polar regions in the centrosymmetric paraelectric phase because it should be zero in the cubic phase with inversion symmetry. These various anomalies associated with the precursor phenomena of SSCG-grown BaTiO_3_ single crystals will be compared to those of high-quality BaTiO_3_ single crystals in order to find out whether the precursor phenomena are common and intrinsic effect irrespective of the crystal quality.

## 2. Results

### 2.1. Dielectric Properties

**Figure 1:**
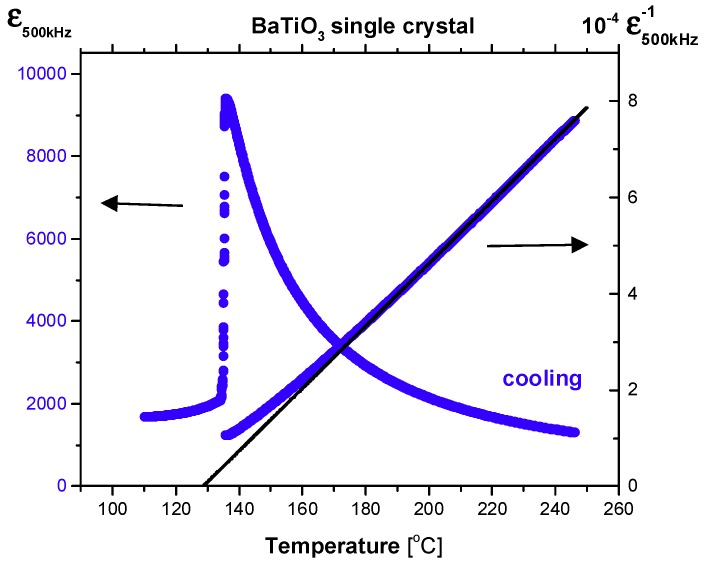
Temperature dependence of the real part of the complex permittivity and its inverse shown on the left and right ordinate, respectively, which was measured at the probe frequency of 500 kHz upon cooling. The solid line is the best-fitted result for the inverse dielectric constant in the paraelectric phase obtained by using the Curie–Weiss law (see the text).

Figure 1 shows the temperature dependence of the real part of the complex dielectric constant, *ε*, measured at the probe frequency of 500 kHz, the scale of which is shown on the left ordinate. The right ordinate denotes the inverse of the permittivity. The permittivity shows a divergent behavior in the paraelectric phase upon cooling toward the Curie point (*T_C_* ≈ 136 °C). The ferroelectric phase transition is clearly perceived from the sharp cusp in the permittivity. The high-temperature data above ≈200 °C is linear and thus can be fitted by using the Curie–Weiss law, which is given by *ε* = *C*/(*T* − *T_CW_*) + *ε*_∞_. Here, *T* is temperature*, C* is the Curie constant, *T_CW_* is the Curie-Weiss temperature, and *ε_∞_* is the high-frequency permittivity, respectively. The linear line in Figure 1 is the best-fitted result obtained by using the Curie–Weiss law resulting in *C* = 1.54 × 10^5^ K, *T_CW_* = 402 K (129 °C), and *ε*_∞_ = 6. The measured dielectric constant exhibits a noticeable deviation from the Curie–Weiss law at temperatures below ≈200 °C consistent with previous results [18]. Especially, the deviation becomes substantial at temperatures below ≈180 °C. Similar dielectric behaviors are common in typical relaxor ferroelectrics such as Pb(Mg_1/3_Nb_2/3_)O_3_ [51]. In the case of relaxors, the permittivity deviates from the Curie–Weiss law at the Burns temperature, *T_B_*, where the PNRs begin to appear. The dipolar interaction among the PNRs is responsible for this deviation. In a similar context, the deviation observed in BaTiO_3_ can be attributed to the precursor polar clusters formed in the paraelectric phase and onset of dipolar interactions among them. The relation between this anomalous behavior and other pre-transitional phenomena will be discussed below in more detail.

### 2.2. Brillouin Scattering Results

#### 2.2.1. Acoustic Phonon Modes

**Figure 2:**
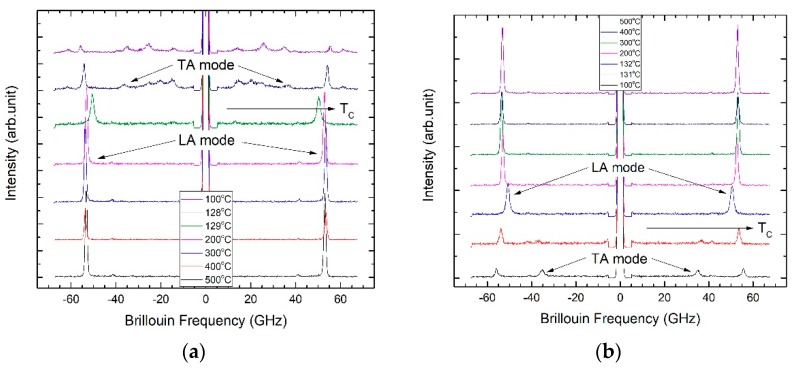
Temperature dependence of the Brillouin spectrum of BaTiO_3_ measured at the backward scattering geometry upon (**a**) cooling and (**b**) heating. The phonon propagation direction was <100>. The LA and the TA mode indicate the longitudinal and the transverse acoustic mode, respectively. The ferroelectric phase transition temperature *T_C_* is indicated by a horizontal arrow.

Figure 2a,b shows the temperature dependence of a Brillouin spectrum of BaTiO_3_ measured at the backward scattering geometry upon cooling and heating, respectively. The frequency range was ±66 GHz (±2.2 cm^−1^), which is suitable for observing Brillouin doublets caused by the acoustic phonon modes. The high-temperature spectra above *T_C_* consist of two Brillouin doublets located at ≈53 GHz and 41 GHz, which correspond to the LA mode and the transverse acoustic (TA) mode, respectively. The phonon propagation direction was <100> in the cubic phase. The LA and the TA modes are related to the elastic constants *C*_11_ and *C*_44_, respectively, in the present scattering geometry. The changes in the mode frequency and the half width of the LA mode are substantial at temperatures near *T_C_*. The TA mode was very weak in the paraelectric phase. In principle, the TA mode was forbidden in the cubic phase according to the Brillouin selection rule [46], and its weak appearance seems to be due to the aperture broadening effect caused by the finite solid angle of the objective lens [17]. In the tetragonal ferroelectric phase, the acoustic modes, especially the TA mode, consisted of multiple peaks as can be seen from the spectra at 100 °C and 128 °C. This was mainly due to the formation of ferroelectric domains, each domain with different orientations contributing to the Brillouin scattering.

**Figure 3:**
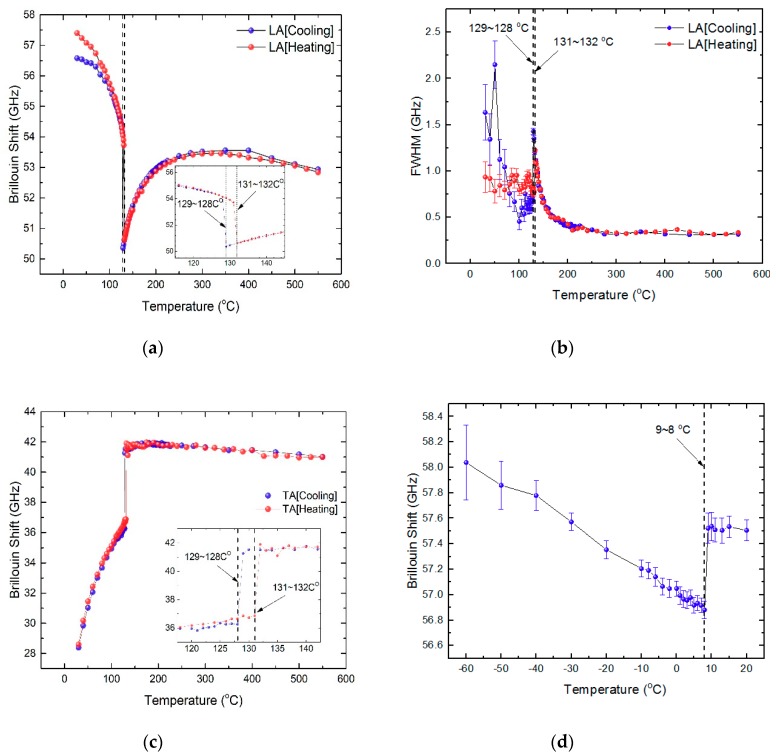
Temperature dependence of (**a**) the Brillouin shift of the LA mode, (**b**) the full width at half maximum (FWHM) of the LA mode, (**c**) the Brillouin shift of the TA mode, and (**d**) the Brillouin shift of the LA mode around the tetragonal–orthorhombic transition point. The two insets in (**a**,**c**) are the extended views of the data in the vicinity of *T_C_*. The temperature ranges given in numbers denote the phase transition temperatures.

The Brillouin spectra were curve-fitted using the Voigt function, which is the convolution of the Lorentzian function and the Gaussian instrumental function. The width of the Gaussian function was fixed to the pre-determined instrumental function of the interferometer. The Brillouin frequency shift (ν_B_) and the full width at half maximum (FWHM, Γ_B_) of the acoustic modes were derived from the curve-fitting procedure as a function of temperature. The ν_B_ and the Γ_B_ are related to the sound velocity and the acoustic attenuation coefficient, respectively. Figure 3a,b show the temperature dependence of the ν_B_ and the Γ_B_, respectively, of the LA mode for both heating and cooling processes. The ν_B_ exhibited a monotonously increasing behavior upon cooling from the highest temperature, reached a maximum at ≈310 °C, and then decreased gradually upon further cooling. The discontinuous change in the ν_B_ in the vicinity of ≈132 °C indicates the ferroelectric phase transition from cubic to tetragonal phase. The inset of Figure 3a shows an extended view of the ν_B_ near *T_C_* demonstrating the existence of thermal hysteresis in *T_C_*, thus revealing the first-order character of the phase transition of BaTiO_3_. The substantial change in the ν_B_ near *T_C_* was accompanied by the increasing Γ_B_, as can be seen in Figure 3b. The Γ_B_ displayed a λ-type anomaly in the paraelectric phase close to *T_C_*. The significant increase in the acoustic attenuation along with the substantial mode softening above *T_C_* has been attributed to the polarization fluctuations in the paraelectric phase of BaTiO_3_ [16,17,44]. The formation of precursor polar clusters with local symmetry breaking (non-centrosymmetric regions) in the ideal cubic phase causes the mode softening and the substantial acoustic damping (or attenuation) in the cubic phase [52].

Figure 3c shows the temperature dependence of the TA mode frequency. It was nearly temperature independent in the paraelectric phase, which means that the elastic constant *C*_44_ did not change appreciably in the cubic phase. The discontinuous change in the TA mode frequency at *T_C_* shows a thermal hysteresis consistent with the LA mode behavior. Figure 3d shows the change in the ν_B_ of the LA mode at low temperatures in the ferroelectric phase. The step-like change near 9 °C corresponds to the tetragonal–orthorhombic phase transition of BaTiO_3_. 

#### 2.2.2. Quasi-Elastic Central Peaks

**Figure 4:**
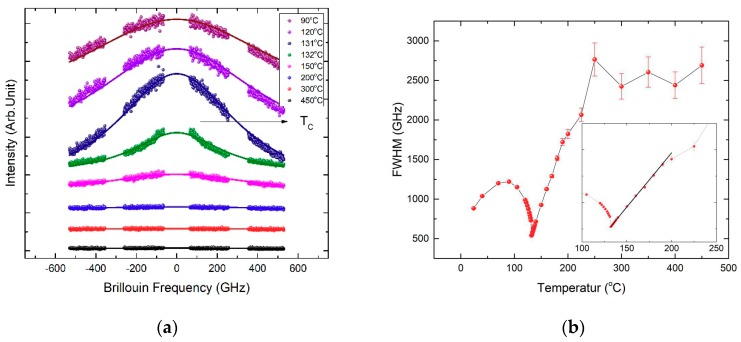
Temperature dependence of (**a**) the depolarized (VH) spectrum and (**b**) the FWHM of BaTiO_3_ measured in a wider frequency range of ±540 GHz (±18 cm^−1^). The inset shows the extended view of the FWHM near *T_C_*. The solid line denotes the fitting result in terms of Equation (1).

Both polarized (VV) and the depolarized (VH) inelastic light scattering spectra of BaTiO_3_ were measured in a wider frequency range of ±540 GHz (±18 cm^−1^), but the intensity of the VH spectrum was stronger than that of the VV spectrum. Thus, we focused on the analysis of the VH spectrum as a function of temperature. Figure 4a shows the temperature dependence of the VH spectrum of BaTiO_3_. The high-temperature spectra were nearly flat without any contribution from the low-frequency Raman modes because the low-lying optic phonons were located far above the present frequency range. In addition, the polarization of the incident beam was oriented long the cubic <110> direction, which is known to suppress the low-frequency Raman optic mode located at ≈50 cm^−1^ [53]. A quasi-elastic central peak grew as temperature decreased, especially as the sample cooled down toward *T_C_*. The intensity of the central peak decreased once the temperature passed *T_C_* and further decreased. The central peak could be fitted in terms of a single Lorentzian function superposed on a constant background with the solid lines in Figure 4a indicating the best-fitted results. It indicates that a single Debye-type relaxation process was responsible for the occurrence of the central peak. Considering the substantial changes in the acoustic phonon modes near *T_C_*, the growth of the central peak was related to the relaxational motion of precursor polar clusters and their growth in size upon cooling toward *T_C_*.

The full width at half maximum of the central peak(Γ_CP_) is inversely proportional to the relaxation time of the polar clusters (*τ*_CP_) via Γ_CP_ = 1/(π*τ*_CP_). It is assumed that there is no relaxation time distribution of the polar clusters, which may be justified at high temperatures above *T_C_* [54]. The obtained temperature dependence of the Γ_CP_ is shown in Figure 4b. The relaxation time grows upon cooling toward *T_C_*, which indicates slowing-down of the relaxation process of the polar clusters. The linear decrease in the Γ_CP_ in the paraelectric phase suggests that the formula describing the critical slowing-down behavior [2] given by Equation (1) may be used to fit the data shown in Figure 4b:

(1) $$\frac{1}{\pi \tau_{CP}}=\frac{1}{\pi \tau_{0}}\frac{T_{0}}{T-T_{0}},$$

where *τ*_0_ and *T*_0_ are fitting parameters. It has often been used to describe the order–disorder phase transition of ferroelectrics [2]. The solid line in Figure 4b denotes the best-fitted result, and the obtained fitting parameters are shown in Table 1. The general behavior of the slowing down process is similar to that of the TSSG-grown BaTiO_3_ [17]. 

### 2.3. Birefringence and Piezo-Response

The birefringence is in principle zero in the ideal centrosymmetric cubic phase of BaTiO_3_. However, local symmetry breaking in the pre-transitional polar clusters and the resulting symmetry lowering may contribute to the birefringence of cubic BaTiO_3_ even though its average symmetry is cubic. The colorless and transparent BaTiO_3_ single crystal grown using the TSSG method exhibited a weak birefringence over a certain temperature range above *T_C_*, which was clear evidence for the existence of non-centrosymmetric polar clusters in the nonpolar cubic phase [17,20].

**Figure 5:**
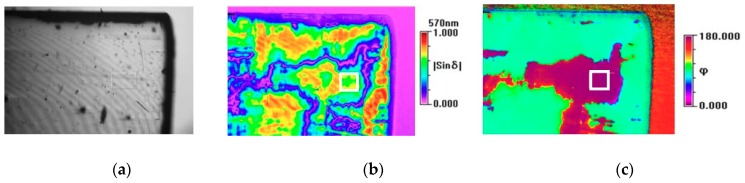
Maps of (**a**) the transmitted light intensity of 570 nm wavelength, (**b**) the |sin*δ*|, and (**c**) the angle φ measured for BaTiO_3_ single crystal at 250 °C, far above *T_C_*. The meaning of each symbol is described in the text. The rectangle visible in Figure (**c**) is the area of 160 × 100 µm^2^ for which the data shown in Figure 6a were calculated. Black points in Figure (**a**) are the air pores visible on the crystal surface.

In the present study, we used a high-precision birefringence imaging system [50] to measure the plano-birefringence of the BaTiO_3_ single crystal grown by the SSCG method. It consisted of a polarizing microscope where the polarizer could be rotated to a fixed angle from a reference position. The intensity measured at a certain position on the crystal is given by the following equation:

(2) $$I=\frac{1}{2}I_{0}+\frac{1}{2}I_{0}\sin\delta \cos2\varphi \sin\tau -\frac{1}{2}I_{0}\sin\delta \sin2\varphi \cos\tau ,$$

where *I*_0_ is a constant value, δ(=2πΔnL/λ) is the phase difference between the two polarized light components, φ is the angle of an axis of the optical indicatrix of the specimen projected onto the image measured from a predetermined direction, and *τ* is *ωt* (*ω* is the angular frequency of the excitation light, and *t* the time.). Regarding *δ*, *Δn* is the plano-birefringence, *L* is the thickness of the sample and *λ* the laser wavelength. Figure 5a–c show false-color images of the intensity, |sin*δ*|, and φ, respectively, measured at 250 °C far above *T_C_*. In principle, the birefringence and the |sin*δ*| should be zero at this temperature if the crystal symmetry is strictly cubic with the center of symmetry. However, the |sin*δ*| and φ in Figure 5 show complex distributions over the sample area which is mainly due to defects, stacking faults, pores, and strains formed during the crystal growth. Some air pores and defects are clearly seen from Figure 5a. The distributions of the |sin*δ*| and φ measured at 250 °C did not change over a temperature range above 200 °C, thus this constant background distribution was subtracted from the birefringence measured at lower temperatures in order to track the net contribution due to the precursor polar clusters.

**Figure 6:**
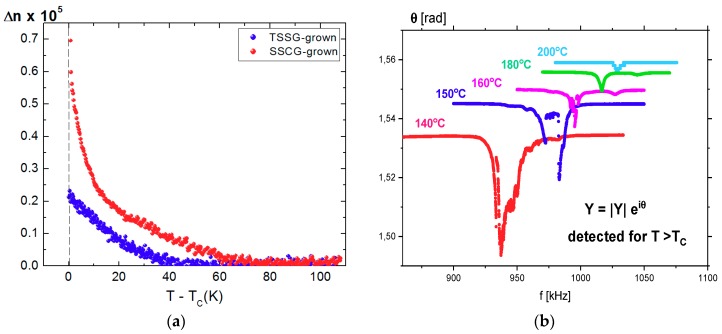
(**a**) Temperature dependence of the birefringence of both BaTiO_3_ single crystals above T_C_. The data of the TSSG-grown BaTiO_3_ was taken from Reference [17]. (**b**) Local minimum of the phase shift of the admittance accompanying piezoelectric resonances measured in the paraelectric phase for the virgin crystal by means of dynamic method and presented at several temperatures.

The small rectangle in Figure 5 is the area of 160 × 100 µm^2^ where both |sin*δ*| and *φ* were relatively homogeneous, and the temperature dependence of the birefringence of this area is shown in Figure 6a. For comparison, the birefringence of the TSSG-grown BaTiO_3_ is also plotted with respect to *T* − *T_C_* [17]. Both crystals show negligible birefringence at temperatures above *T_C_* + 60 °C and increasing behavior upon cooling. The reason for the growing birefringence on approaching *T_C_* is that sizes of polar regions grow with decreasing temperature. The closer to *T_C_*, the bigger the sizes of these regions were. Since these regions were non-centrosymmetric, the difference between the refractive indexes of polar regions grew, leading to an increase in birefringence. Moreover, they can locally self-organize themselves, i.e., they become ordered and have a higher polarity. Birefringence values of both crystals show similar temperature dependences and are of similar order of magnitude (≈10^−5^) in spite of the significant difference in the optical quality and level of defects of the two crystals. The non-zero value of the birefringence clearly indicates the existence of the non-centrosymmetric polar clusters embedded in the cubic phase of BaTiO_3_. Moreover, the fact that the colorless TSSG-grown single crystal and the yellow-colored SSCG-grown single crystal caused by defects included during technological process exhibits birefringence of similar values suggests that the observed birefringence and the related precursor phenomena are intrinsic properties irrespectively of the defect levels.

In order to corroborate the existence of the non-centrosymmetric polar regions in the paraelectric phase via an additional method, the piezoelectric activity of the sample was investigated by observing the piezoelectric resonances using a dynamic method described in Reference [55]. The total admittance *Y* flowing through the sample is expressed as |*Y*|e*^i^^θ^*, where |*Y*| is the amplitude of the admittance, and *θ* is the phase shift between the voltage applied to the sample and measured |*Y*|. Figure 6b shows the frequency dependence of the *θ* at several temperatures above *T_C_*. It was striking that the piezoelectric activity above *T_C_* was observed for samples without prior poling caused by the d.c. electric field. This means that above *T_C_*, the polar regions existing in the paraelectric matrix could be large enough and ordered such that it gave a macroscopic piezoelectric effect. In the vicinity of the ideal piezoelectric resonance, the *θ* changes with frequency from the 1.57 rad (90°) to −1.57 rad (−90°), and then to 1.57 rad (90°), revealing a local minimum at *θ* equal to −1.57 rad (−90°). It meant that, on the one hand, the dip in *θ* in Figure 6b was clearly related to the appearance of piezoelectric activity in the sample; on the other hand, the very small changes in *θ* point to a very weak piezoelectric effect. However, the gradual disappearance of the dip in *θ* in Figure 6b and irregular runs of *θ*(*f*) suggest that the piezoelectric effect in the paraelectric phase of BaTiO_3_ came from the ordered polar regions interacting through the elastic paraelectric matrix, and became weaker while temperature rose. No dip in the *θ(f)* was observed at temperatures above 200 °C, which is consistent with the disappearance of the birefringence in the same temperature range.

## 3. Discussion

All physical properties investigated in this study show anomalous behaviors in the paraelectric phase of BaTiO_3_ single crystals grown using the SSCG method. The dielectric constant exhibits deviation from the Curie–Weiss law at temperatures below 200 °C. In the same temperature range, weak but clear piezoelectric signals and birefringence values were observed as shown in Figure 6. These results undoubtedly indicate the existence of non-centrosymmetric local regions because the birefringence and piezoelectricity should disappear in the centrosymmetric cubic phase. It also consists with the observation of the second harmonic generation signal in the cubic phase, which should be zero in a centrosymmetric system [24]. These regions of broken local symmetry were not static because the two properties grow in their intensities on approaching *T_C_*. These non-centrosymmetric clusters should be polar as they affect the permittivity. The enhancement of dipolar interactions among the polar clusters near *T_C_* would decrease the permittivity, which is indeed observed as shown in Figure 1. Moreover, relaxational motions of the polar clusters induce quasi-elastic central peaks in the inelastic light scattering spectrum (see Figure 4). These results are consistent with this suggestion and other numerous results reporting precursor phenomena of BaTiO_3_ in the paraelectric phase [4,5,6,7,8,9,10,11,12,13,14,15,16,17,18,19,20,21,22,23,24,25,26,27,28,31,32,33].

A nuclear magnetic resonance (NMR) study revealed that Ti off-centered motions are correlated to form nano-sized clusters in the paraelectric phase of BaTiO_3_ [8,9]. These correlated clusters have their inherent polarizations that would fluctuate resulting in no net macroscopic polarization. However, local, random polarizations exist and may couple to other physical properties. The longitudinal acoustic mode probed using Brillouin spectroscopy showed softening in its mode frequency, as shown in Figure 3a. The softening became more substantial with the decrease in temperature below 200 °C, which was accompanied by the significant increase in the half width of the LA mode. Electrostrictive coupling between the squared polarization and the strain caused by the LA waves is known to cause the decrease in the relevant sound velocity and the elastic constant [52]. In addition, polarization fluctuations in the paraelectric phase decreases the mode frequency and induces fluctuations damping near *T_C_* [52]. We cannot, however, exclude the possibility of piezoelectric coupling between the polarization in the polar clusters and the strain, especially near *T_C_*, because the piezoelectric activity persists in the temperature range of *T_C_* ~ 200 °C and the dynamics of the polar clusters become sluggish enough to assure the linear coupling between the polarization and the strain [44]. 

It is interesting to note that the TA mode frequency does not change appreciably in the paraelectric phase. There are three eigen elastic constants in the cubic phase, which are *C*_11_ + 2*C*_12_, *C*_11_ − *C*_12_, and *C*_44_. Among them, *C*_11_ − *C*_12_ and *C*_44_ correspond to tetragonal (or orthorhombic) and rhombohedral distortion, respectively [56]. The nearly constant *C*_44_ indicates that the structural instability induced by polar clusters was not rhombohedral. It may rather be tetragonal considering the low-temperature tetragonal phase below *T_C_*. This suggestion is supported by the NMR result where the Ti off-centered motions are correlated to form tetragonal nano-domains [9]. Therefore, we expect the elastic eigenvalue *C*_11_ − *C*_12_ may exhibit significant softening in the paraelectric phase, which should be confirmed by additional acoustic study. 

The most interesting result of this study is that the overall precursor phenomena of SSCG-grown BaTiO_3_ single crystals are similar to those of the TSSG-grown ones. As Figure 5 demonstrates, the SSCG-grown BaTiO_3_ single crystals exhibit inherent extended defects, impurities, and air pores due to the crystal growth condition where no melting process is included [41,42]. In spite of the static birefringence caused by these various faults of the SSCG-grown BaTiO_3_ single crystal, the birefringence caused by the precursor polar clusters exhibits nearly the same behavior to that of the TSSG-grown BaTiO_3_. In order to corroborate this suggestion, the dielectric permittivity and the LA mode properties were compared between the SSCG-grown and the TSSG-grown BaTiO_3_, which were shown in supplementary materials. The inverse of the dielectric permittivity exhibited the deviation from the Curie–Weiss law in nearly the same temperature range in both crystals. Moreover, the mode frequency and the half width of the LA mode of both crystals were indistinguishable from each other in the paraelectric phase. Grabovsky et al. investigated the specific heat of several BaTiO_3_ single crystals of different origin and found that the addition contribution to the specific heat exists in the paraelectric phase [57]. This anomalous high-temperature specific-heat existed up to *T_C_* + 60 K and was independent of the sample growth conditions. They attributed this universal behavior to the contribution from thermal fluctuations of the order parameter instead to the defect contributions. This study combined with the present results clearly suggests that the precursor phenomena of both BaTiO_3_ single crystals are associated with the order parameter fluctuations of precursor polar clusters and not strongly sensitive to the defect/impurity conditions.

Finally, we would like to indicate two points. First, the difference in the birefringence between the two crystals becomes larger upon cooling toward *T_C_*. In the SSCG-grown single crystals, the density of defects was higher than in the TSSG-grown one. These defects may be extended defects of a much more complex geometrical structure, such as dislocations, rather than point defects. In the vicinity of such defects, a field of local stresses appears and produces unit cell deformation. The paraelectric matrix was highly polarizable and thus this stress field may induce additional polar regions, giving a contribution to the macroscopic birefringence. Second, although the present study unambiguously showed the universal behavior of precursor polar clusters irrespective of the crystal condition, the exact nature of these pretransitional regions need to be revealed at an atomic scale, which needs a more sophisticated approach, such as X-ray absorption near-edge structure measurements, in order to look into the change in local structures [58].

## 4. Materials and Methods

### 4.1. Materials

**Figure 7:**
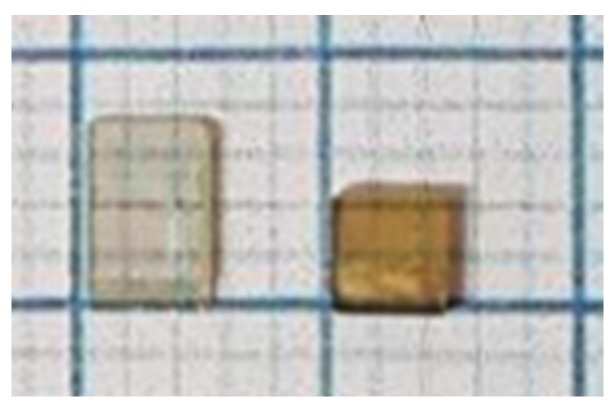
The left and the right single crystals were grown in terms of TSSG and SSCG method, respectively. The TSSG-grown BaTiO_3_ single crystal was investigated in Reference [17].

The BaTiO_3_ single crystals were grown using the solid-state crystal growth method [41,42]. Raw powders of BaCO_3_ and TiO_2_ were used to prepare polycrystalline ceramics via a general sintering process. After weighting the raw materials at the stoichiometric ratio, they were mixed for 24 h using a ball-milling process. It was calcined at 1000 °C, dried, and then subjected to the second ball-milling. It was then compressed into a ceramic plate form, dried, and then sintered. A small seed BaTiO_3_ crystal was placed on the sintered body of the polycrystalline ceramics. The second heat treatment was carried out for 100 h for SSCG of BaTiO_3_ single crystal. In this process, the BaTiO_3_ seed crystal was continuously grown into the BaTiO_3_ single crystal larger than 25 mm × 25 mm × 5 mm. There was no melting process during the crystal growth, which resulted in no compositional gradient and high chemical homogeneity. The grown single crystals were cut into (100) platelets in the cubic coordinates and polished to optical quality. Figure 7 shows the grown single crystal in terms of the present SSCG method (right) along with the crystal grown by using the TSSG method (left). The extended view of the intensity of the plano-birefringence, as shown in Figure 5a, reveals pores and extended defects in the crystal.

### 4.2. Methods

The crystal was put in a compact temperature controller (THMSE600, Linkam, Tadworth, UK), which was combined with a modified microscope (BX-41, Olympus, Tokyo, Japan) for backscattering measurements. The Brillouin spectrum was measured using a conventional tandem six-pass Fabry-Perot interferometer (TFP-2, JRS Co., Zürich, Switzerland) and a diode-pumped solid-state laser (Excelsior 532-300, Spectra Physics, Santa Clara, CA, USA). The wavelength of the laser was 532 nm operated at the power of 16 mW. The free spectral range of the interferometer was set to be 75 GHz and 300 GHz for probing the acoustic modes and central peaks, respectively. A conventional photon-counting system combined with a multichannel analyzer (1024 channels) was used to detect and average the signal. The details of the experimental setup can be found elsewhere [59,60]. 

The birefringence was measured in terms of a birefringence imaging system (Metripol, Oxford Cryosystems, Oxford, UK). The sample was inserted in a high-precision temperature stage (TMSG600, Linkam, Tadworth, UK) where the temperature could be maintained within 0.1 K. The temperature was changed at a rate of 0.2 K/min. The Metripol system consisted of a polarizing microscope and a CCD camera. The microscope was equipped with a plane-polarizer, which could be rotated by means of a computer interface. The detailed description of this technique can be found elsewhere [50]. The dielectric and piezoelectric properties were investigated using the standard dielectric spectroscopy and dynamical method for piezoelectric activity, respectively. Dielectric measurements were performed at a temperature rate not faster than 1 K/min, but the piezoelectric response was measured at constant temperature through the measurement of admittance in a function of frequency.

## Figures and Tables

**Table 1:** The best-fitted parameters of BaTiO_3_ single crystals by using Equation (1).

Fitted *T* Range	$1/\pi \tau_{0}\;(\mathrm{GHz})$	*T*_0_ (°C)
≈*T_C_*–*T_C_* + 60 °C	7374	103

## Supplementary Materials

The following are available online. Figure S1: temperature dependence of the real part of the complex permittivity and its inverse of the SSCG-grown and TSSG-grown BaTiO_3_ single crystals. Figure S2: temperature dependence of the Brillouin shift of the LA mode and the FWHM of the LA mode of both TSSG-grown and SSCG-grown BaTiO_3_ single crystals.
