# Supplementary material for: Precursor Phenomena of Barium Titanate Single Crystals Grown Using a Solid-State Single Crystal Growth Method Studied with Inelastic Brillouin Light Scattering and Birefringence Measurements
Source: Molecules. 2018 Dec 1;23(12):3171. doi: 10.3390/molecules23123171 (PMC6321232; doi:10.3390/molecules23123171)
Supplement: Supplementary file 1 [file molecules-23-03171-s001.pdf]

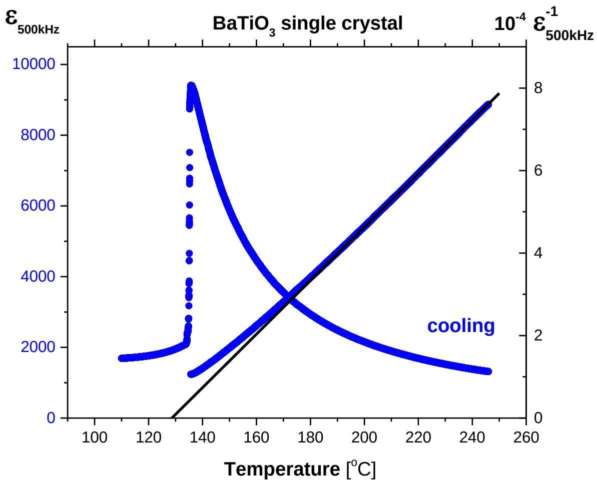

(**a**)

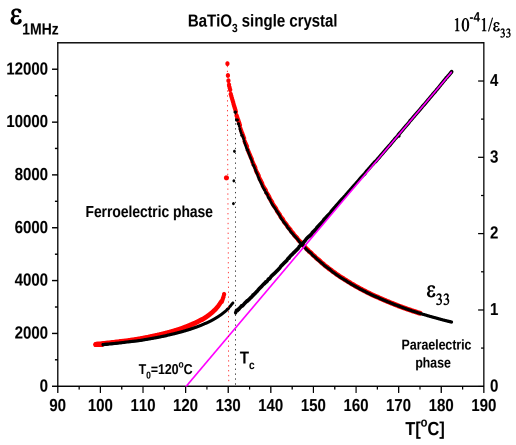

(**b**)

Figure S1. Temperature dependence of the real part of the complex permittivity and its inverse shown on the left and right ordinate, respectively, of the (**a**) SSCG-grown and (**b**) TSSG-grown $BaTiO_3$ single crystals. The solid line is the best-fitted result for the inverse dielectric constant in the paraelectric phase obtained by using the Curie–Weiss law. The data of TSSG-grown $BaTiO_3$ were the modified one reported in Reference 18.

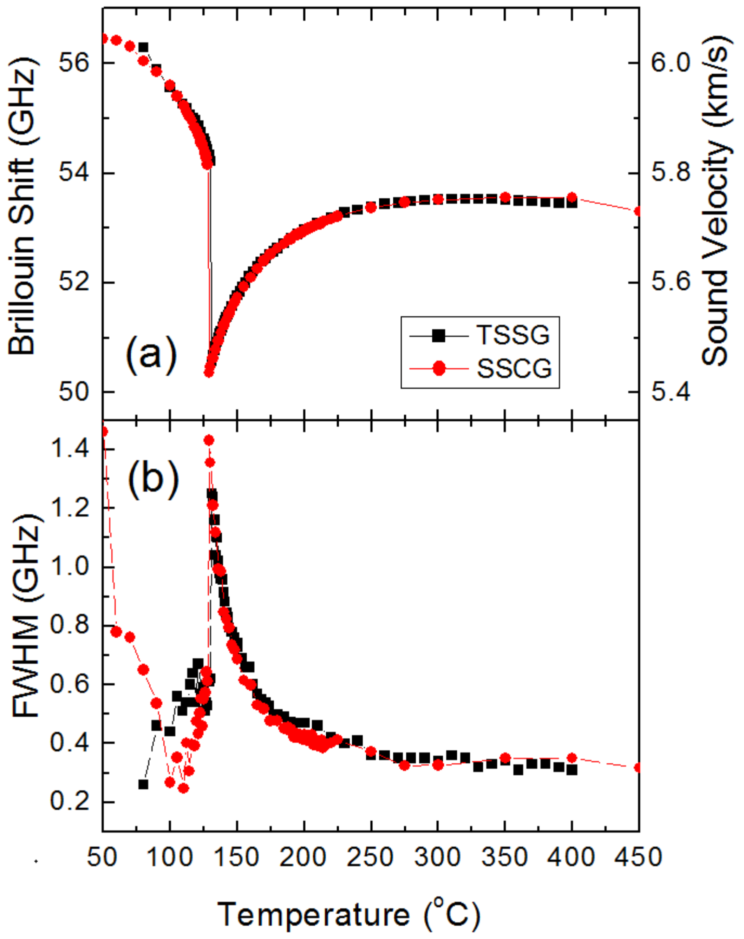

Figure S2. Temperature dependence of (**a**) the Brillouin shift of the LA mode, (**b**) the FWHM of the LA mode of both TSSG-grown and SSCG-grown $BaTiO_3$ single crystals. The mode frequency and the FWHM of the TSSG-grown $BaTiO_3$ were modified from the reported values in Reference 17.
